# Radiomic analysis of pathologically confirmed mandibular osteoradionecrosis to identify cases with associated recurrence: a multicentric retrospective study

**DOI:** 10.1016/j.phro.2026.101031

**Published:** 2026-07-03

**Authors:** Olivier Ozcan, Remy Laluc, Aline Carsin-Vue, Nathaniel Assouly, Esteban Brenet, Antonio Da Silva Ribeiro Mota, Stephane Derruau, Marine Fontaine, Camille Invernizzi, Oriane Marques, Nicolas Passat, Arnaud Beddok

**Affiliations:** a Department of Radiation Oncology, Institut Godinot, Reims, France; b Department of Informatics, Institut Godinot, Reims, France; c Department of Pediatric Radiology, CHU Reims, Reims, France; d Department of Surgery, Institut Godinot, Reims, France; e Department of Head and Neck Surgery, CHU Reims, Reims, France; f Department of Oral Surgery, CHU Reims, Reims, France; g Department of Radiation Oncology, ICONE, Reims, France; h University of Reims Champagne-Ardenne, CRESTIC, Reims, France

**Keywords:** Osteoradionecrosis, Recurrence, Radiation Therapy, Surgery, Computed Tomography, Radiomics, Pathology

## Abstract

Differentiating mandibular osteoradionecrosis (ORN) from tumor recurrence remains challenging on preoperative imaging. We conducted a multicenter retrospective study including 24 patients with histologically confirmed ORN who underwent surgery and had preoperative CT available. Radiomic features were extracted using an IBSI-compliant pipeline and compared between pure ORN (*n* = 21) and ORN with recurrence (*n* = 3). Three features showed nominal uncorrected differences between groups. Given the rarity of histologically confirmed recurrence, this study was designed as an exploratory hypothesis-generating analysis. These findings suggest that CT radiomics may capture subtle imaging differences and warrant validation in larger cohorts.

## Highlights

• Differentiating mandibular osteoradionecrosis from recurrence remains difficult.
• No radiomic studies have addressed this question to date.
• CT radiomics analyzed in pathologically confirmed osteoradionecrosis cases.
• Radiomics may support preoperative identification of recurrence.

## Graphical abstract

**Figure:**
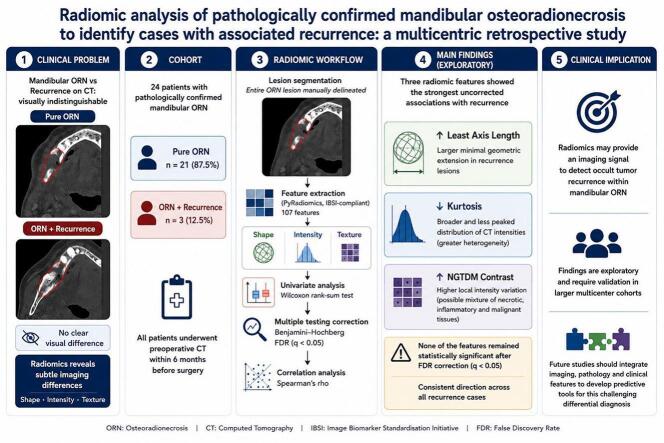

## 1 Introduction

Osteoradionecrosis (ORN) is a severe late complication of radiotherapy for head and neck cancer, with contemporary studies reporting incidences generally ranging from 3% to 6%, depending on the definition used and the population studied [1], [2]. Although ORN is commonly diagnosed based on clinical and imaging findings, a subset of patients presents with tumor recurrence developing within or adjacent to the necrotic bone. Distinguishing pure ORN from ORN associated with recurrent malignancy remains a major diagnostic challenge, even with contrast-enhanced computed tomography (CT) or FDG PET/CT, as both conditions may exhibit overlapping radiological and metabolic features.

Accurate preoperative identification of recurrence is clinically critical. Surgical management of ORN can be extensive, often requiring segmental mandibulectomy with substantial functional and aesthetic morbidity. Misclassification of tumor recurrence as ORN may delay appropriate oncologic management, whereas over-suspicion of recurrence may expose patients to unnecessary diagnostic procedures or overtreatment. Improving the diagnostic performance of preoperative imaging, therefore, represents an important unmet need.

Conventional imaging modalities have limitations in this context. While solid or cystic soft-tissue masses may favor tumor recurrence, and bony sclerosis is more suggestive of ORN, substantial overlap exists, particularly on FDG PET/CT, where inflammatory and malignant processes may show similar metabolic activity [3]. Accordingly, the American Society of Clinical Oncology 2024 guideline recommends that ORN diagnosis be primarily based on clinical and radiographic findings and does not routinely endorse biopsy because of the risk of exacerbating necrosis, while emphasizing that distinguishing ORN from synchronous malignancy remains essential for patient management [4]. More recently, Watson et al. proposed the ClinRad classification, a risk-based staging system designed to standardize the assessment of ORN severity and facilitate clinical management and research [5].

Recent studies have further shown that occult cancer may be present in a non-negligible proportion of advanced ORN cases, with identified risk factors including clinical bleeding, early surgical intervention, and extensive disease burden, underscoring the need for diagnostic vigilance [6]. However, no imaging or radiomic signature has yet been validated to reliably distinguish pure ORN from ORN harboring tumor recurrence in pathologically confirmed cohorts.

Radiomics, through the extraction of high-dimensional quantitative features from medical images, has emerged as a promising approach to characterize imaging phenotypes beyond visual assessment. In head and neck oncology, radiomic features have been associated with tumor biology, treatment response, and recurrence patterns [7], [8]. While CT radiomics has recently been investigated for differentiating mandibular ORN from healthy bone, its potential role in identifying occult recurrence within histologically confirmed mandibular ORN remains unexplored.

The present multicenter retrospective study, therefore, aims to explore whether radiomic features extracted from preoperative CT scans are associated with the presence of tumor recurrence within histologically proven mandibular ORN. This work does not aim to develop or validate a predictive model, but rather to determine whether a radiomic signal exists in this rare and clinically challenging scenario, and to inform the design of future multicenter cohorts.

## 2 Materials and methods

### 2.1 Study population

This retrospective study included 24 consecutive patients with histologically confirmed mandibular osteoradionecrosis (ORN) treated between 2019 and 2024 at three institutions (Institut Godinot, CHU Reims, and ICONE). All patients underwent surgery for ORN and had a preoperative CT scan available for analysis. Inclusion criteria included: (1) pathologic confirmation of ORN on surgical specimens; (2) availability of a preoperative CT scan of the mandible; and (3) availability of digital imaging data suitable for radiomic feature extraction. Patients were classified into two groups based on pathologic assessment: pure ORN (*n* = 21) and ORN with associated tumor recurrence (ORN + Recurrence; *n* = 3). The study was specifically designed to investigate occult tumor recurrence within surgically treated ORN lesions; therefore, patients with isolated mandibular recurrence without histological evidence of ORN were not included. This study complied with the French MR-004 regulatory framework of the National Commission on Informatics and Liberty (CNIL) for research not involving human participants (RNIPH 3). The study was registered under the MR-004 reference methodology (Dossier No. 29367751).

### 2.2 CT acquisition and image preprocessing

All CT scans covered the face and mandible and were performed according to institutional protocols (tube voltage 70–130 kVp, tube current 4–533 mAs, slice thickness 0.6–2.0 mm; see **Supplementary Table 1** for acquisition details). Image preprocessing followed the recommendations of the Image Biomarker Standardization Initiative (IBSI) [9]. No intensity normalization or absolute rescaling was applied before feature extraction. All image volumes were resampled to an isotropic voxel spacing of 1 × 1 × 1 mm^3^ using B-spline interpolation to minimize variability related to acquisition geometry. Segmentation masks were resampled using nearest-neighbor interpolation to preserve label integrity. Intensity discretization was performed using a fixed bin width of 25 Hounsfield units (HU), as recommended for CT-based radiomic analyses. This discretization strategy was applied consistently across all images without imposing a fixed number of gray levels.

### 2.3 Delineation of ORN volumes

**Fig. 1:**
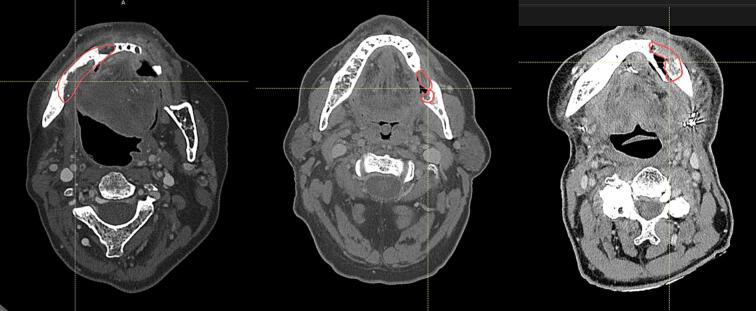
**Manual delineation of mandibular osteoradionecrosis on preoperative CT.** Representative axial CT slices illustrating the segmentation of osteoradionecrosis volumes (red contours). The contours encompass necrotic bone, bone sequestra, and surrounding inflammatory tissue as visible on CT. These segmentations were used for subsequent radiomic analysis. (For interpretation of the references to colour in this figure legend, the reader is referred to the web version of this article.)

The ORN regions were manually contoured on axial CT images by a radiation oncologist and independently reviewed by an experienced head-and-neck radiologist blinded to the radiomic analysis. The delineated volume encompassed all radiological manifestations of osteoradionecrosis, including necrotic mandibular bone, bone sequestra, fistulous tracts, and adjacent inflammatory soft tissue when present (Fig. 1). The radiologist review aimed to ensure consistency of lesion delineation and identify imaging findings suggestive of associated tumor recurrence. No separate contour was generated for the recurrence component. In patients classified as ORN + Recurrence, tumor recurrence was identified only on histopathological examination of the surgical specimen and could not be reliably distinguished from ORN on preoperative CT. Therefore, a single ROI encompassing the entire lesion was delineated in all patients, irrespective of the final pathological diagnosis.

### 2.4 Radiomic feature extraction

Radiomic feature extraction was performed using a standardized Python-based workflow compliant with the IBSI recommendations and implemented with the PyRadiomics library. A total of 107 radiomic features were extracted. These included 14 shape-based features describing the three-dimensional geometric properties of the regions of interest, 18 first-order intensity histogram features characterizing the distribution of voxel intensities within the segmented volumes, and 75 texture features. The texture features were derived from the gray-level co-occurrence matrix (GLCM, *n* = 24), gray-level run length matrix (GLRLM, *n* = 16), gray-level size zone matrix (GLSZM, n = 16), gray-level dependence matrix (GLDM, *n* = 14), and neighboring gray-tone difference matrix (NGTDM, *n* = 5). All feature definitions strictly adhered to IBSI nomenclature and mathematical formulations, ensuring methodological consistency and reproducibility [9].

### 2.5 Statistical analysis

Group comparisons were performed using the Wilcoxon rank-sum test, with *p* < 0.05 considered statistically significant. As a sensitivity analysis, *p*-values were additionally adjusted for multiple comparisons using the Benjamini-Hochberg false discovery rate (FDR) procedure. Features reaching significance were further evaluated using Spearman correlation analysis to assess redundancy, with a threshold of ρ ≥ 0.70. An exploratory bootstrap resampling analysis (1000 iterations) was performed. Given the very limited number of recurrence cases, this analysis should be interpreted cautiously and not considered a formal assessment of statistical robustness. As an additional sensitivity analysis, a leave-one-out analysis was performed across the three recurrence cases. For each candidate feature, the analysis was repeated after exclusion of each recurrence case in turn to assess whether the direction of the observed association was dependent on a single patient. Because of the limited number of recurrence cases, multivariable modeling or classification analyses were not attempted. All analyses were performed using Python version 3.10.6 and R version 4.3.1.

## 3 Results

### 3.1 Patient characteristics

The study population consisted of 24 patients with histologically confirmed mandibular ORN (**Supplementary Table 2**). The median age was 69.5 years (IQR: 63.75–72.5), and the majority of patients were male (20/24, 83%). The most frequent primary tumor site was the oral cavity (12 patients, 50%), followed by the oropharynx (9 patients, 37.5%), with only a minority of tumors arising from the hypopharynx or larynx (1 patient, 4.2%) or other sites (2 patients, 8.3%). Squamous cell carcinoma was the predominant histological subtype (22 patients, 92%), with only two patients presenting with other histologies. Initial RT was delivered using intensity-modulated RT (IMRT) in 22 patients, while the radiation technique was unavailable for two patients. Concurrent chemotherapy was administered in 15 patients (62.5%). The median interval between RT and ORN diagnosis was 15.4 months. Pathological examination identified pure ORN in 21 patients (87.5%) and ORN associated with tumor recurrence in 3 patients (12.5%). The median interval between CT acquisition and surgery was 86 days (IQR 45–200).

### 3.2 Radiomic feature differences between groups

**Fig. 2:**
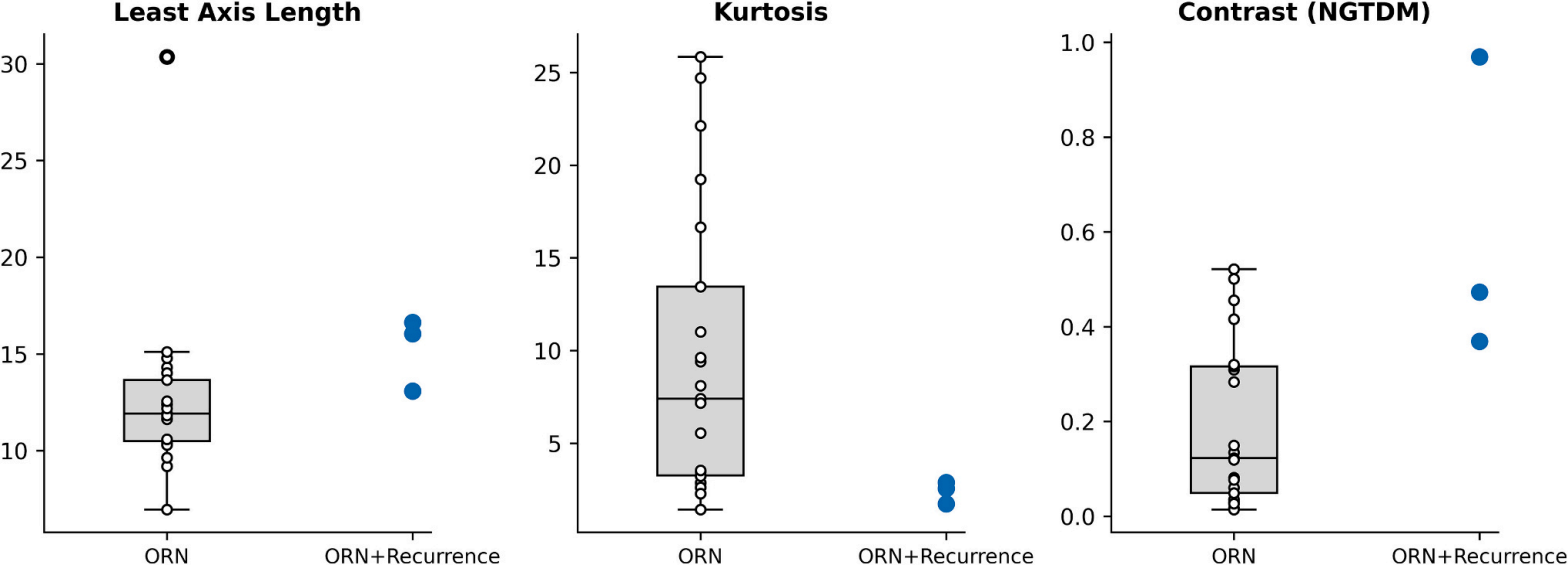
**Radiomic features showing the strongest uncorrected associations with recurrence.** Individual data points are shown for both groups. Boxplots are displayed only for the pure ORN group (*n* = 21), whereas ORN + Recurrence cases (*n* = 3) are shown as individual points only. *P*-values were calculated using the Wilcoxon rank-sum test. None of the associations remained statistically significant after Benjamini-Hochberg correction.

Three radiomic features showed the strongest uncorrected associations with recurrence and showed nominal uncorrected differences between pure ORN and ORN + Recurrence (Fig. 2). Least Axis Length showed a median value of 11.92 mm (IQR: 10.49–13.65) in pure ORN and 16.04 mm (IQR: 14.56–16.33) in ORN + Recurrence (*p* = 0.041). Kurtosis had a median of 7.41 (IQR: 3.26–13.45) in pure ORN and 2.56 (IQR: 2.15–2.73) in ORN + Recurrence (*p* = 0.031). NGTDM Contrast had a median of 0.12 (IQR: 0.05–0.32) in pure ORN and 0.47 (IQR: 0.42–0.72) in ORN + Recurrence (*p* = 0.023). None of these associations remained statistically significant after Benjamini-Hochberg correction for multiple testing (all FDR-adjusted *p*-values = 0.535). Individual feature values for the three recurrence cases are reported in **Supplementary Table 3**. All recurrence cases exhibited higher Least Axis Length and NGTDM Contrast values, and lower Kurtosis values, than the median values observed in the pure ORN group. In leave-one-out analyses of the three recurrence cases, the direction of these associations remained unchanged for all three candidate features, suggesting that the results were not driven by a single outlier case (**Supplementary Table 4**). Pairwise correlations between these three features are shown in Supplementary Fig. 1. All Spearman correlation coefficients were below 0.7, with the highest absolute correlation observed between Kurtosis and NGTDM Contrast (ρ = −0.63).

### 3.3 Exploratory bootstrap resampling analysis

Bootstrap resampling suggested that the direction of the observed effects remained generally consistent across resamples for all three features. However, the upper quantiles of the bootstrap *p*-value distributions exceeded 0.05, indicating that statistical significance was not consistently preserved across resamples, as expected given the very small number of recurrence cases (*n* = 3).

## 4 Discussion

In this exploratory analysis of 24 patients with pathologically confirmed ORN, three CT-derived radiomic features, Least Axis Length, intensity Kurtosis, and NGTDM Contrast, showed the strongest uncorrected associations with recurrence. Lesions with recurrence exhibited a larger minimal geometric extension, lower intensity kurtosis, and higher local contrast than pure ORN lesions. Although none of these associations remained statistically significant after correction for multiple testing, the direction of the observed effects was consistent across the three recurrence cases and in leave-one-out analyses, suggesting potential structural and attenuation differences within the affected mandibular region. While the biological meaning of individual radiomic features remains uncertain, some hypotheses may explain these observations. Higher Least Axis Length values may reflect a greater minimal spatial extent of lesions harboring recurrence. Lower Kurtosis values suggest a broader and less peaked distribution of CT intensities, potentially reflecting increased tissue heterogeneity. Similarly, higher NGTDM Contrast values indicate greater local intensity variation, which may be consistent with the coexistence of necrotic, inflammatory, and malignant tissue components within the same lesion. These interpretations remain speculative and should be considered hypothesis-generating.

The coexistence of ORN and tumor recurrence was rare in this cohort, with only three cases identified. Consequently, the limited number of events precluded the development of conventional machine-learning models and restricted the analysis to exploratory comparisons of individual radiomic features. The absence of statistically significant associations after correction for multiple testing further underscores the exploratory nature of these findings. Nevertheless, the observed differences across geometric and texture features suggest potential imaging differences between pure ORN and ORN associated with recurrence that warrant evaluation in larger cohorts. These findings should therefore be interpreted as hypothesis-generating rather than confirmatory.

The difficulty of differentiating osteoradionecrosis from tumor recurrence has been highlighted in prior literature. Cheng et al. developed a PET/CT-based scoring system in 103 patients with suspected mandibular ORN after treatment for oral squamous cell carcinoma [10]. In their analysis, younger age, a soft-tissue–predominant SUVmax location, and high total lesion glycolysis (TLG, defined as the product of metabolic tumor volume and mean standardized uptake value) greater than 62.68 g were identified as independent predictors of mandibular recurrence. A three-point clinical–imaging score achieved a sensitivity of 87.5% and a specificity of 82.3% for identifying recurrence. These findings highlight the potential value of quantitative imaging biomarkers in this challenging differential diagnosis.

The present study complements this approach by showing that structural CT radiomic features may also provide discriminatory information within clinically and radiographically ambiguous ORN regions. Together, these findings suggest that integrative approaches combining clinical variables, PET metabolic parameters, CT radiomics, and treatment-related data may improve diagnostic accuracy in this setting.

Beyond PET-based biomarkers, other imaging modalities have also been investigated to characterize radiation-induced mandibular damage. Dynamic contrast-enhanced MRI (DCE-MRI) has been shown to detect vascular alterations associated with osteoradionecrosis. In a prospective study including patients with advanced ORN, Mohamed et al. reported significantly increased vascular permeability parameters such as Ktrans and Ve in ORN regions compared with contralateral healthy mandible, reflecting radiation-induced microvascular damage [11]. Although these findings suggest that DCE-MRI may help identify vascular alterations associated with ORN, its role in differentiating osteoradionecrosis from tumor recurrence has not yet been specifically evaluated.

Finally, the relatively short interval between imaging and surgical management observed in this cohort (median 86 days) reflects the clinical work-up of suspected osteoradionecrosis and the need for timely surgical intervention in patients with progressive mandibular damage. In this context, imaging performed during this diagnostic window may represent a critical opportunity to identify radiological features suggestive of tumor recurrence before surgical confirmation.

This study has several limitations. First, it is retrospective and includes a limited number of recurrence cases, reducing statistical power and preventing robust multivariable modeling. Second, no formal interobserver reproducibility analysis of the segmentations was performed. Although all contours were reviewed by an experienced head-and-neck radiologist, the potential impact of segmentation variability on radiomic feature values cannot be excluded. Third, although all examinations were CT-based, imaging acquisition protocols were not fully standardized across centers. Fourth, only CT data were analyzed, whereas multimodal imaging approaches incorporating PET or MRI may provide additional discriminatory information. Despite these limitations, the study has several strengths. All cases were pathologically confirmed, providing a reliable reference standard for the diagnosis of ORN and recurrence. In addition, the radiomic workflow followed a standardized pipeline compliant with IBSI recommendations, and the analysis addressed a well-defined clinical question that is highly relevant for surgical decision-making. Future studies integrating multimodal imaging and larger multicenter cohorts will be required to validate these preliminary findings and assess the potential clinical utility of radiomics in this diagnostic setting.

This exploratory study provides initial evidence that CT radiomic features may differ between pure ORN and ORN associated with tumor recurrence. Although further validation in larger multicenter cohorts is required, these findings support the development of collaborative datasets integrating imaging, pathological, and clinical features. Recent initiatives aimed at standardizing ORN assessment and reporting, including the ClinRad classification [5] and the international Delphi consensus defining minimum data elements for ORN research [12], may provide an important framework for future multicenter radiomic studies and predictive modeling efforts in this setting.

## Data Sharing Statement

Research data are stored in an institutional repository and will be shared upon request to the corresponding author.

## CRediT authorship contribution statement

**Olivier Ozcan:** Writing – original draft, Visualization, Investigation, Data curation, Conceptualization. **Remy Laluc:** Writing – review & editing, Software, Data curation. **Aline Carsin-Vue:** Writing – review & editing, Visualization, Validation, Investigation. **Nathaniel Assouly:** Writing – review & editing, Investigation. **Esteban Brenet:** Writing – review & editing, Investigation. **Antonio Da Silva Ribeiro Mota:** Writing – review & editing, Investigation. **Stephane Derruau:** Writing – review & editing, Investigation. **Marine Fontaine:** Writing – review & editing, Investigation. **Camille Invernizzi:** Writing – review & editing, Investigation. **Oriane Marques:** Writing – review & editing, Investigation. **Nicolas Passat:** Writing – review & editing, Methodology. **Arnaud Beddok:** Writing – review & editing, Visualization, Validation, Supervision, Project administration, Investigation, Formal analysis, Conceptualization.

## Declaration of competing interest

The authors declare that they have no known competing financial interests or personal relationships that could have appeared to influence the work reported in this paper.

## Appendix A Supplementary data

Supplementary material 1 Sup Figure 1. Correlation heatmap of the three significant radiomic features (Spearman’s coefficients).
