# Supplementary material for: Radiomic analysis of pathologically confirmed mandibular osteoradionecrosis to identify cases with associated recurrence: a multicentric retrospective study
Source: Phys Imaging Radiat Oncol. 2026 Jul 3;40:101031. doi: 10.1016/j.phro.2026.101031 (PMC13449477; doi:10.1016/j.phro.2026.101031)
Supplement: Supplementary file 1 [file mmc1.docx]

**Sup Figure 1.** **Correlation heatmap of the three significant radiomic features (Spearman’s coefficients).**

All pairwise correlations were below the exclusion threshold of 0.7, confirming that Least Axis Length, Kurtosis, and NGTDM Contrast carried non-redundant information. The strongest correlation was observed between Kurtosis and NGTDM Contrast (ρ = –0.63).
