# Supplementary material for: Radiomic analysis of pathologically confirmed mandibular osteoradionecrosis to identify cases with associated recurrence: a multicentric retrospective study
Source: Phys Imaging Radiat Oncol. 2026 Jul 3;40:101031. doi: 10.1016/j.phro.2026.101031 (PMC13449477; doi:10.1016/j.phro.2026.101031)
Supplement: Supplementary file 2 [file mmc2.docx]

| **Parameter** | **Value** |
| --- | --- |
| **Number of patients** | 24 |
| **CT manufacturer** | GE Healthcare (n = 8), Philips (n = 10), Siemens Healthineers (n = 6) |
| **CT scanner models** | GE Revolution EVO, GE Discovery 710; Philips Ingenuity Flex, Ingenuity TF PET/CT; Siemens SOMATOM go.Top, SOMATOM Scope, Biograph Vision |
| **Tube voltage (kVp)** | 70–130 (median: 120) |
| **Tube current (mAs)** | 4–533 (median: 170) |
| **Slice thickness (mm)** | 0.625–2.0 (most frequent: 1.5) |
| **Reconstruction matrix** | 512 × 512 (all scans) |
| **Pixel spacing (mm)** | 0.37–0.98 |
| **Field of view (mm)** | 189–500 |
| **Reconstruction kernel** | Bone / high-resolution (e.g. BONE, BONEPLUS, YA, Br60f, Hr40f, I80s) |
| **Acquisition mode** | Helical (all available cases) |
| **Pitch** | 0.51–1.45 |

**Supplementary Table 1.** CT acquisition parameters

**Supplementary Table 2.** Baseline characteristics of the 24 patients with histologically confirmed mandibular ORN

| **Characteristics** | **Value** |
| --- | --- |
| **Age (years), median [IQR]** | 69.5 [63.75–72.5] |
| **Sex** |  |
| Male | 20 (83%) |
| Female | 4 (17%) |
| **Primary tumor site** |  |
| Oral cavity | 12 (50%) |
| Oropharynx | 9 (37.5%) |
| Hypopharynx / larynx | 1 (4.2%) |
| Other | 2 (8.3%) |
| **Histology of initial tumor** |  |
| Squamous cell carcinoma | 22 (92%) |
| Other | 2 (8%) |
| **Initial radiation technique** |  |
| IMRT / VMAT | 22/24 |
| NA | 2/24 |
| **Concurrent chemotherapy during initial RT** |  |
| Yes | 15 (62.5%) |
| No | 9 (37.5%) |
| **Interval from RT to ORN onset (months)** | 15.4 (median) |
| **Type of ORN presentation** |  |
| Pure ORN | 21 (87.5%) |
| ORN + recurrence | 3 (12.5%) |
| **Preoperative imaging** |  |
| Contrast-enhanced CT (all patients) | 24 (100%) |

**Supplementary Table 3.** Individual radiomic feature values in the three ORN+Recurrence cases

| **Case** | **Least Axis Length (mm)** | **Kurtosis** | **NGTDM Contrast** |
| --- | --- | --- | --- |
| ORN+Recurrence #1 | 16.04 | 2.56 | 0.970 |
| ORN+Recurrence #2 | 16.58 | 1.73 | 0.369 |
| ORN+Recurrence #3 | 13.10 | 2.89 | 0.473 |

**Supplementary Table 4.** Leave-one-out sensitivity analysis of the three recurrence cases

| **Feature** | **ORN median** | **Excluding case 1** | **Excluding case 2** | **Excluding case 3** |
| --- | --- | --- | --- | --- |
| Least Axis Length | 11.92 | 14.85 | 14.56 | 16.33 |
| Kurtosis | 7.41 | 2.31 | 2.73 | 2.15 |
| NGTDM Contrast | 0.12 | 0.42 | 0.72 | 0.67 |
